# The Importance of Structured Scientific Enquiry: A priority for the MEAJO Editorial Board

**DOI:** 10.4103/0974-9233.53861

**Published:** 2009

**Authors:** Rajiv Khandekar

**Affiliations:** Eye & Ear Health Care, Ministry of Health Oman and Hon attaché Physician, BCEIO, University of British Columbia, Vancouver, BC, Canada

It is an honour and pleasure to write an editorial for the Middle East African Journal of Ophthalmology (MEAJO).

The editorial board of MEAJO met during the MEACO meeting in Bahrain and expressed their commitment to ensure the journal publishes high quality manuscripts that fulfil the scope of the journal. This is indeed a big challenge and a joint responsibility of the ophthalmologists in the region to conduct high quality scientific research and share their work through publications. The editorial board will extend limited assistance in English editing. However, the submitted manuscripts will henceforth undergo strict scientific peer review paying close attention to methods to abide international guidelines for scientific publications.

Many ophthalmic institutions now include evidence based medicine as part of the undergraduate and postgraduate curricula. Even during the process of accreditation and promotion of ophthalmologists in these institutions, considerable importance is given to research and publications. We encourage all budding scientists and ophthalmologists therefore to properly plan research studies and publish the results of their research in MEAJO. This journal has a wide readership in Asian and African continents. Recently the journal has been made available online. Thus articles can be easily accessible to a wider audience without cost.

During the MEACO meeting, societies for subspecialties in ophthalmology were formed. A theme issue for each subspecialty will be published in MEAJO. This will give a platform for the experts in the region and internationally to share their scientific work and express expert views.

Clinical ophthalmology keeps the ophthalmic practioner busy. However, to improve their own skills, they should also introspect, raise scientific queries and get evidence based answers.^1^ Sharing experiences through publications will certainly benefit other clinicians working in remote areas of this subcontinent who are also looking for answers for preferred practices suitable to the local conditions.

Obtaining clinically relevant information from attending international meetings is widely popular among ophthalmologists. This is an important activity for an ophthalmogist to improve their skills. However, the presentations are often influenced by observer bias. In addition, presentations do not undergo critical peer review and hence their content should be carefully interpreted and adopted only after the data appears in peer reviewed journals.

Those keen on publishing their research in good journals must pay attention to ethical, epidemiological and statistical aspects of their studies especially during the planning stage. One should understand that randomized clinical trials and longitudinal studies provide evidence that are superior in quality when compared to case reports and case series.^2^ Collaborative work with a team of clinicians, statisticians, epidemiologists and field staff at different stages of a study pays rich dividends in making the publication robust. A manuscript is worthy if it has a clear message for practising ophthalmologists and policy makers. They will help in formulating or alter public health policies and improve eye care in the community.

The articles in this edition cover a variety of subjects. In the VISION 2020 initiative uncorrected refractive error and low vision is a priority within the disease control strategy.^3^ A consultation on the integration of eye care within school health was held during MEACO 2009. Special attention was given to uncorrected refractive error and eye care for children of school going age.

Padhye *et al*. in their review article compared refractive error and other eye problems in 13 to 15 years old urban and rural students in a state of India. The prevalence of myopia was higher in students of urban schools. They also suggested that the strategies for screening and intervention should differ in rural compared to urban schools.

The baseline and one year follow up information on children with low vision disability that were given low vision optical aids was authored by Sabra *et al*. It is interesting to note that all fifty children had impaired contrast sensitivity. This observation needs further studies and if found consistent, intervention by environmental changes as well as providing non-optical aids could benefit them.^4^

Diabetes is a known risk factor that affects visual outcome and recovery following cataract surgery.^5^ The article from Nigeria by Onakpoya *et al*. confirmed this observation. If poor and good visual outcomes are grouped and odds of having diabetes or non diabetes in these two outcomes were studied, it would fit the description of a case control study. Here patients with cataract were grouped as those with diabetes and those without diabetes. They were followed after cataract surgeries and visual outcomes were evaluated. Hence this seems more like a cohort study.

Waziri-Erameh *et al*. reviewed hospital based data of five years and analysed the present pattern of eye diseases seen in a hospital of Nigeria. Although visual acuity and causes of impairment are presented, they may not represent a catchment area and hence formulation of public health approach on its basis will be difficult.

It is evident that all authors have sincerely undertaken research to the best of their capacity and published the outcomes. However, I encourage the authors to use the pilot data published in this issue to conduct additional focused studies with input from epidemiologists and statisticians that would create studies that would have a significant impact on eye care in the region.
